# Correlation of hemorrhage, axonal damage, and blood-tissue barrier disruption in brain and retina of Malawian children with fatal cerebral malaria

**DOI:** 10.3389/fcimb.2015.00018

**Published:** 2015-03-16

**Authors:** Jesse Greiner, Katerina Dorovini-Zis, Terrie E. Taylor, Malcolm E. Molyneux, Nicholas A. V. Beare, Steve Kamiza, Valerie A. White

**Affiliations:** ^1^Department of Pathology and Laboratory Medicine, Vancouver General Hospital and University of British ColumbiaVancouver, BC, Canada; ^2^College of Medicine, University of MalawiBlantyre, Malawi; ^3^Blantyre Malaria ProjectBlantyre, Malawi; ^4^Department of Osteopathic Medical Specialities, Michigan State UniversityEast Lansing, MI, USA; ^5^Malawi-Liverpool-Wellcome Trust Clinical Research ProgrammeBlantyre, Malawi; ^6^Department of Pathology, Malawi College of MedicineBlantyre, Malawi; ^7^Liverpool School of Tropical MedicineLiverpool, UK; ^8^Department of Eye and Vision Science, University of LiverpoolLiverpool, UK; ^9^St. Paul's Eye Unit, Royal Liverpool University Hospital, LiverpoolLiverpool, UK; ^10^Department of Ophthalmology and Visual Science, Vancouver General Hospital and University of British ColumbiaVancouver, BC, Canada

**Keywords:** cerebral malaria, malaria retinopathy, sequestration, hemorrhages, permeability, axonal damage

## Abstract

**Background:** The retinal and brain histopathological findings in children who died from cerebral malaria (CM) have been recently described. Similar changes occur in both structures, but the findings have not been directly compared in the same patients. In this study, we compared clinical retinal findings and retinal and cerebral histopathological changes in a series of patients in Blantyre, Malawi, who died of CM.

**Methods:** The features systematically compared in the same patient were: (1) clinical, gross and microscopic retinal hemorrhages with microscopic cerebral hemorrhages, (2) retinal and cerebral hemorrhage-associated and -unassociated axonal damage, and fibrinogen leakage, and (3) differences in the above features between the pathological categories of CM without microvascular pathology (CM1) and CM with microvascular pathology (CM2) in retina and brain.

**Results:** Forty-seven patients were included: seven CM1, 28 CM2, and 12 controls. In the 35 malaria cases retinal and cerebral pathology correlated in all features except for non-hemorrhage associated fibrinogen leakage. Regarding CM1 and CM2 cases, the only differences were in the proportion of patients with hemorrhage-associated cerebral pathology, and this was expected, based on the definitions of CM1 and CM2. The retina did not show this difference. Non-hemorrhage associated pathology was similar for the two groups.

**Comment:** As postulated, histopathological features of hemorrhages, axonal damage and non-hemorrhage associated fibrinogen leakage correlated in the retina and brain of individual patients, although the difference in hemorrhages between the CM1 and CM2 groups was not consistently observed in the retina. These results help to underpin the utility of ophthalmoscopic examination and fundus findings to help in diagnosis and assessment of cerebral malaria patients, but may not help in distinguishing between CM1 and CM2 patients during life.

## Introduction

Cerebral malaria (CM) is the most serious manifestation of *Plasmodium falciparum (P. falciparum)* infection occurring in ~1% of all infected individuals and carrying a fatality rate of 15–20%, resulting in up to 675,000 deaths per year (World Health Organization, 2010). Children under the age of five in sub-Saharan Africa account for 90% of CM-related deaths. The clinical diagnosis of CM is based on the presence of deep coma often associated with seizures in the presence of *P. falciparum* parasitemia and in the absence of other sufficient explanations for coma. In Malawian children diagnosed with cerebral malaria, the post mortem pathologic changes in the brain (Dorovini-Zis et al., 2011) and retina (Lewallen et al., 1999; White et al., 2009) have been described. The contribution of the observed pathological features to the pathogenesis of this disease is at present poorly defined.

Accurate diagnosis of cerebral malaria during life is often challenging owing to the high prevalence of asymptomatic parasitemia in African children (Smith et al., 1993; Mwangi et al., 2005) and the non-specific nature of the main manifestations of the disease (English et al., 1996; Kallander et al., 2004). In an autopsy study of Malawian children with clinically defined CM, 7/31 (23%) of the patients showed no pathological evidence of CM and death was attributed to other causes (Taylor et al., 2004). Distinctive retinal changes in CM that are visible by ophthalmoscopy during life include retinal whitening and orange or white discoloration of retinal vessels; retinal hemorrhages are also common, and papilledema is seen in some patients (Beare et al., 2006; Lewallen et al., 2008). “Malaria retinopathy” is recognized as a sensitive and specific indicator of cerebral involvement in *P. falciparum* infection (Beare et al., 2006; Lewallen et al., 2008; Birbeck et al., 2010) and can strengthen the accuracy of a clinical diagnosis of CM (Beare et al., 2011). Including ante-mortem malaria retinopathy as a diagnostic criterion for cerebral malaria improved specificity in a series of fatal cases from 61 to 100% (Beare et al., 2004, 2006; Taylor et al., 2004). Examination of the retina provides a unique opportunity to directly observe, in living patients, a tissue that develops embryologically from the central nervous system. A recent detailed study described the pathological correlates of malaria retinopathy in 35 Malawian children with autopsy confirmed CM, 13 children with the clinical diagnosis of CM but with a non-malarial cause of death, and 16 age-matched children who died of coma of other causes (White et al., 2009). All CM cases showed prominent infected red blood cell (iRBC) sequestration in retinal microvessels, which was associated with gross and microscopic hemorrhages and presence of fibrin-platelet thrombi in ~70% of the cases, with axonal damage in ~50% and with disruption of the blood-retinal barrier (BRB) in ~30%.

In a recent prospective autopsy study we described the salient neuropathological features of CM in the brains of 50 Malawian children who fulfilled the clinical criteria of CM during life (Dorovini-Zis et al., 2011). Of the 50 children, 37 had prominent iRBC sequestration and no other cause of coma, and 13 had non-malarial causes of death and little or no intracerebral sequestration. The cases with prominent microvascular iRBC sequestration could be subdivided into two groups based on the presence or absence of associated microvascular pathology. The group with sequestration in the absence of vasculopathic changes was designated CM1 and the group with sequestration and intra- and perivascular pathology, CM2. In the CM2 group, sequestration was associated with perivascular ring hemorrhages, microvascular thrombosis, disruption of the blood-brain barrier (BBB), and intravascular accumulation of hemozoin-containing monocytes. The perivascular changes consisted of areas of myelin and axonal damage around ring hemorrhages and thrombosed microvessels. Foci of diffuse myelin and axonal damage in areas with prominent iRBC sequestration, unrelated to ring hemorrhages were present in both CM1 and CM2 cases, albeit with greater frequency and severity in the CM2 cases.

Although other work describing, in detail, the retinal pathology of cerebral malaria outlines similar pathological features as those in the brain (Looareesuwan et al., 1983; Lewallen et al., 1999; Schemann et al., 2002; White et al., 2009), a direct comparison of the histopathology of brain and retina in the same patients in order to assess potential similarities and differences between the two has not been previously performed. For the present study we postulated that, owing to their common embryological development from the neuroectoderm, the pathological features present in the two tissues would positively correlate. We therefore looked for correlations between the brain and retina for: (1) presence of sequestration; (2) presence and number of hemorrhages; (3) presence and extent of axonal damage, and (4) presence and degree of blood-tissue barrier disruption. We also compared these features in retina and brain of patients classified as having CM1 or CM2 pathology.

## Methods

Ethical approval was obtained for the study from the ethics committees of the College of Medicine, University of Malawi, Blantyre, Malawi; Michigan State University, East Lansing, MI, USA; Liverpool School of Tropical Medicine, UK; and The University of British Columbia, Vancouver, Canada.

### Patients

The patients included 47 Malawian children with clinically defined cerebral malaria (*P. falciparum* parasitemia, a Blantyre coma score of ≤2/5 at 30 min after treatment of hypoglycemia and/or seizures, and exclusion of other identifiable causes of coma including bacterial meningitis) who died of the disease between 1996 and 2003. Informed consent to undergo autopsy was received from each of the families (Taylor et al., 2004).

The cases were divided into three groups based on the post-mortem microscopic examination of the brain as previously described (Taylor et al., 2004; Dorovini-Zis et al., 2011). The first group, CM1, exhibited only sequestration of iRBC within the microvasculature of the brain. The second group, CM2, demonstrated iRBC sequestration with additional microvascular pathology including intravascular microthrombi and ring hemorrhages surrounding areas of necrosis. The third (control) group, CM3, lacked evidence of iRBC sequestration and non-malarial causes of death were identified in all cases. The final pathological diagnoses of the CM3 group of patients included pneumonia (4), viral pneumonitis (1), possible severe pneumonia (1), Reye's syndrome (2), Klebsiella bacteremia and HIV infection (1), hepatic necrosis (1), severe anemia (1), and ruptured arteriovenous malformation (1).

### Protocol

Patients were cared for on a dedicated research ward; antimalarials were provided according to national treatment guidelines and clinical management was as described previously (Taylor et al., 2004). Experienced ophthalmologists carried out ophthalmological funduscopic examinations shortly after admission to hospital via both direct and indirect ophthalmoscopy on dilated pupils. The results were recorded on standardized forms as previously described (Harding et al., 2006). Autopsies were performed using a standardized dissection and collection protocol consisting of examination, specimen photography, weighing, and sectioning in the fresh state (Taylor et al., 2004; Dorovini-Zis et al., 2011).

### Eyes

After removal, eyes were fixed in 10% formalin and opened in the pupil-optic nerve plane with the superior calotte removed (White et al., 2009). Under the dissecting microscope hemorrhages were quantified similar to the clinical examination (Grade 1: 1–5, Grade 2: 5–20, Grade 3: 20–50, and Grade 4: >50 hemorrhages) (Harding et al., 2006). The pupil-optic nerve block was processed through graded alcohols, xylene, and embedded in paraffin wax as per standard protocols. Sections were cut at 3 μm and stained with hematoxylin and eosin (H&E) or Periodic-Acid-Schiff (PAS), or used for immunohistochemistry. Microscopic hemorrhages were semi-quantitatively evaluated from one to four by the number of quadrants of the retina that contained at least one hemorrhage when viewed after sectioning the globe through the pupil-optic nerve plane.

### Brain

Gross cerebral hemorrhages were assessed at the time of autopsy as being present or absent. Representative blocks were taken from the frontal and parietal lobes of the brain, fixed in formalin and embedded in paraffin wax. Six μm thick sections were stained with H&E and Luxol fast blue/H&E. Microscopically ring hemorrhages were quantified by counting the number of hemorrhages per 10 randomly selected fields under a Nikon Labophot light microscope at 10X magnification using a 1 cm^2^ ocular grid.

### Immunohistochemistry

Sections, 3 μm thick, were stained with the indirect immunoperoxidase technique for β-Amyloid Precursor Protein (β-APP; 22C11, 1:500, mouse monoclonal; Chemicon, Temecula, CA) to detect axonal damage, and for fibrinogen (1:500, rabbit polyclonal; Dako, Carpinteria, CA) to assess BRB and BBB disruption as previously described (Dorovini-Zis et al., 2011).

Quantification of axonal damage in both retina and brain was done by counting the number of foci with β-APP positive axons that were hemorrhage-associated (HA β-APP) or non-hemorrhage associated (NHA β-APP) in 10 randomly selected fields in each section at 10X magnification. The size of the foci was measured using a 1 cm^2^ ocular grid at 10X magnification and expressed as μm^2^ area of axonal damage. Quantification of fibrinogen leakage was carried out by counting the areas of focal fibrinogen staining both associated and not associated with hemorrhages per 10 randomly selected fields at 10X magnification.

### Statistical analysis

Variables included clinical ophthalmoscopic, gross pathologic and microscopic retinal hemorrhages; microscopic foci of hemorrhage associated (HA), non-hemorrhage associated (NHA), and area of β-APP staining; and microscopic foci of HA and NHA fibrinogen extravasation. Fisher's exact test was used to evaluate categorical values, which included the numbers of patients positive for each of the variables in the retina vs. the brain in the entire group. We calculated correlations between retinal and brain sections across all subjects for each variable using Spearman's correlation coefficient. Mann-Whitney U tests were done to detect differences between CM1 and CM2 cases in all variables.

## Results

**Figure 1:**
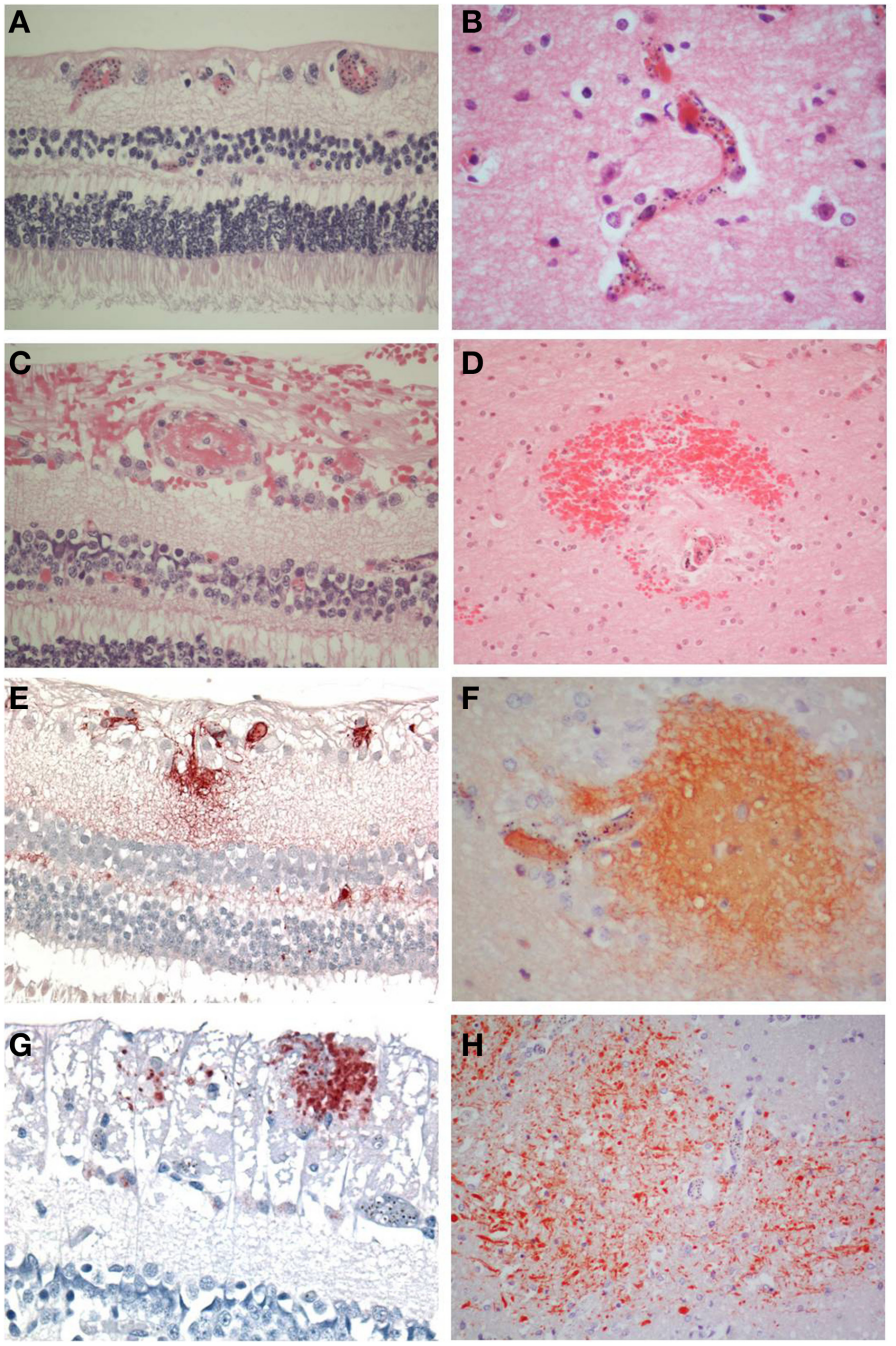
**Comparisons of the salient pathological findings in the retina and brain of children with CM**. **(A)** Sequestration of late stage malaria parasites in post-capillary venules of the ganglion cell layer and capillaries of the inner nuclear layer and **(B)** in the cerebral cortex. **(C)** A thrombus containing vessel in the retina surrounded by hemorrhage is the equivalent of **(D)** a ring hemorrhage in the cerebral white matter with a central thrombosed microvessel and hemozoin granules. **(E)** Fibrinogen extravasation in the inner retina and **(F)** in the brain is associated with prominent iRBC sequestration in the absence of hemorrhage. **(G)** Axonal damage, indicated by β-APP immunoreactive abnormal axons, in the ganglion cell layer of the retina in the absence of hemorrhage. **(H)** Diffuse axonal damage in the cerebral white matter in the form of irregular patches of β-APP positive tortuous and swollen axons in areas of heavy iRBC sequestration. **A–D**: H&E; **E, F**: fibrinogen; **G, H**: β-APP. **(A–C, E, F, H)** x400; **(D, H)** x200.

There were 47 patients in total, seven patients classified as CM1, 28 as CM2, and 12 as CM3. The clinical and demographic characteristics have been previously described (Dorovini-Zis et al., 2011) (Table 1). In the retina and brain of both CM1 and CM2 patients, the lumen of most microvessels contained sequestered iRBC and variable numbers of malaria pigment (hemozoin) granules (Figures 1A,B) as previously described (Taylor et al., 2004; Dorovini-Zis et al., 2011; Barrera et al., 2014). In the CM2 group, sequestration was often associated with thrombosis of the microvessel and perivascular hemorrhages.

**Table 1:** **Numbers of patients with the listed features over the total number of patients examined for each feature**.

	**CM1**		**CM2**		**CM3**		**Total**	
	**Retina**	**Brain**	**Retina**	**Brain**	**Retina**	**Brain**	**Retina**	**Brain**
**HEMORRHAGES**								
Clinical (ophthalmoscopic) examination	1/5	NA	7/13	NA	0/8	NA	8/26	NA
Gross pathologic examination	2/6	0/7	21/26	21/28	0/12	0/12	23/44	21/47
Microscopic examination	4/7	1/7	21/28	19/28	2/12	0/12	27/47	20/47
**β-APP AXONAL DAMAGE**								
HA	0/7	1/7	9/28	20/28	0/12	1/12	9/47	22/47
NHA	3/7	5/7	14/28	23/28	2/12	1/12	19/47	29/47
**FIBRINOGEN LEAKAGE**								
HA	4/7	0/7	19/28	20/28	1/12	0/12	24/47	20/47
NHA	4/7	7/7	7/28	28/28	1/12	10/12	12/47	45/47

*NA, not applicable; HA, hemorrhage associated; NHA, non-hemorrhage associated*.

### Pathology

Retinal hemorrhages were present in any retinal layer, but tended to be in the inner layers. Occasionally a microvessel in the center of the hemorrhage was visible and contained a thrombus (Figure 1C). The hemorrhages around the vessels were not as sharply defined as in the brain where perivascular ring hemorrhages, consisting of a disrupted and often thrombosed capillary or venule surrounded by an area of necrosis (Figure 1D), were most prevalent in the cerebral white matter. These were a feature of CM2, but not CM1 cases, (as classified on brain pathology), but in the retina, this distinction was not obvious.

### Retinal vs. brain pathology (entire group)

Table 1 displays the number of patients in the three groups with the feature examined. Table 2 shows the means and standard deviations for the counts of the variables in retina and brain as well as the correlation coefficients. Within the entire group of 47 patients, there was an association in individual patients between the presence or absence of clinically observed retinal hemorrhages in the left eye with the microscopic evaluation of brain hemorrhages (*p* = 0.02, Fishers exact test), gross retinal pathologic hemorrhages with microscopic brain hemorrhages (*p* = 0.001) and microscopic retinal hemorrhages with microscopic brain hemorrhages (*p* = 0.02). The clinical evaluation of hemorrhages in the right eye did not quite reach significance (*p* = 0.06). There was also an association between patients with HA β-APP damage in the retina and brain (*p* = 0.003, Fishers exact test), but the association with NHA β-APP did not quite reach significance (*p* = 0.06). Similarly, there was an association between HA fibrinogen leakage (*p* = 0.001), but not between NHA fibrinogen leakage (*p* = 1.000).

**Table 2:** **Means and standard deviations of counts and correlation of pathological changes in the retina and brain in CM patients (all cases)**.

**Variable studied**	**Retina (mean)**	**Retina (sd)**	**Brain (mean)**	**Brain (sd)**	**Spearman correlation coefficient**	***p*-Value**
Clinical hemorrhages R eye^*^	0.83	1.15	NA	NA	0.46	0.02
Clinical hemorrhages L eye^*^	0.61	0.98	NA	NA	0.48	0.01
Gross retinal hemorrhages^*^	1.66	1.4	NA	NA	0.67	<0.001
Microscopic hemorrhages	1.74	1.63	0.58	0.82	0.55	<0.001
HA β-APP foci	0.6	1.52	0.65	1	0.48	0.001
NHA β-APP foci	0.94	1.21	0.56	0.42	0.3	0.04
Area of β-APP foci	1.31	1.65	4.7	4.8	0.38	0.01
HA fibrinogen foci	2.53	3.27	0.63	1.04	0.58	<0.001
NHA fibrinogen foci	1.2	2.61	39.63	14.58	0.12	0.43

*NA, not applicable; HA, hemorrhage associated; NHA, non-hemorrhage associated*.

* *These values are being compared with microscopic brain hemorrhages*.

**Figure 2:**
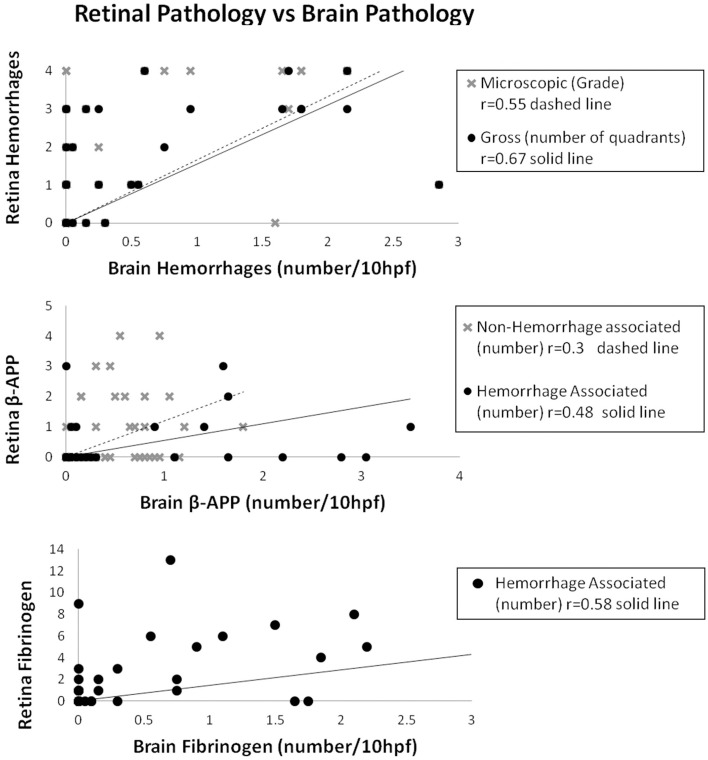
**Correlations of retinal pathology with brain pathology for hemorrhages (top), axonal damage (middle) and HA fibrinogen leakage (bottom)**.

For the entire group, the number of ring hemorrhages per unit area in the brain positively correlated with the semi-quantitative evaluation of numbers of clinical ophthalmoscopic retinal hemorrhages in the right (*r* = 0.46; *p* = 0.02) and left (*r* = 0.48; *p* = 0.01) eyes; as well as with gross pathologic retinal hemorrhages (*r* = 0.67; *p* < 0.001), and microscopic retinal hemorrhages (*r* = 0.55; *p* < 0.001) (Table 2, Figure 2).

The vascular changes in the CM2 group were associated with disruption of the BBB documented by the extravasation of fibrinogen, a 340 kDa serum protein that is normally prevented from entering the brain by the intact BBB. Fibrinogen leakage in the brain and retina was present most often in association with hemorrhages and thrombosis in the CM2 patients. In addition, in both CM1 and CM2 patients, fibrinogen extravasation was also observed around blood vessels with prominent iRBC sequestration in the absence of local hemorrhage (Figures 1E,F). The number of hemorrhage-associated foci of fibrinogen extravasation in the brain positively correlated with the number of hemorrhage-associated areas of fibrinogen extravasation in the retina (*r* = 0.58; *p* < 0.001). However, there was no association between the number of areas of fibrinogen leakage that were non-hemorrhage associated in the brain and retina (*r* = 0.12; *p* = 0.43) with the brain having many more foci of non-hemorrhage associated fibrinogen leakage than the retina.

Axonal damage, as assessed by the presence of strongly β-APP immunoreactive swollen and disrupted axons was seen in the ganglion cell layer of the retina associated or unassociated with hemorrhages (Figure 1G). Axonal changes were seen predominantly in the cerebral white matter in association with ring hemorrhages or as diffuse, irregular and ill-defined focal collections of β-APP positive axons in close proximity to microvessels with prominent iRBC sequestration (Figure 1H) or thrombosis. The ring hemorrhage-associated axonal changes were confined to the CM2 group, whereas both CM1 and CM2 cases showed diffuse axonal damage, although both the number and size of these lesions were greater in CM2 cases. The number of hemorrhage-associated β-APP positive axonal lesions in the brain positively correlated with the number of similar lesions in the retina (*r* = 0.48; *p* = 0.001). A similar positive correlation between the retina and brain was observed for the number of non-hemorrhage associated diffuse axonal lesions (*r* = 0.3; *p* = 0.04) and for the area (*r* = 0.38; *p* = 0.01) of β-APP staining.

### CM1 and CM2 classifications, retina vs. brain

Table 3 shows the means and standard deviations of the variables divided into CM1 and CM2 types. By definition, there was a significant difference between the CM1 and CM2 groups in the median number of hemorrhages in the brain (*p* = 0.008) and this was also observed for gross retinal hemorrhages (*p* = 0.04). This significant difference was not observed for clinical or microscopic retinal hemorrhages. There was no significant difference between the CM1 and CM2 groups in the number of non-hemorrhage associated foci of axonal damage in the retina or brain or in the area of β-APP positive foci in the retina or brain. The difference between the CM1 and CM2 groups in the number of hemorrhage-associated β-APP foci of axonal damage reached significance in the brain (where *p* = 0.05) but not in the retina (*p* = 0.09). There was no significant difference in the number of vessels with fibrinogen leakage between the CM1 and CM2 groups in the retina that were hemorrhage associated or non-hemorrhage associated. In the brain, the difference in number of fibrinogen leakage foci between the CM1 and CM2 groups was significant in hemorrhage associated (*p* = 0.003) but not in non-hemorrhage associated (*p* = 0.28) foci. In summary, there was a significant difference between the CM1 and CM2 groups in all hemorrhage-associated pathology in the brain, but not in the retina. For the non-hemorrhage associated pathology of β-APP staining and fibrinogen deposition, there were no differences between the CM1 and CM2 groups in either the retina or brain.

**Table 3:** **Means and standard deviations of counts in CM1 and CM2 patients and *p*-values for differences between CM1 and CM2**.

**Variable Studied**	**Retina**		**Brain**		Differences^*^	
	**CM1 mean (±SD)**	**CM2 mean (±SD)**	**CM1 mean (±SD)**	**CM2 mean (±SD)**	**Retina (*p*-value)**	**Brain (*p*-value)**
Clinical hemorrhages Rt eye	0.2 (±0.45)	1 (±1.16)	NA	NA	0.16	NA
Gross hemorrhages (grade)	0.67 (±1.21)	1.88 (±1.36)	NA	NA	**0.04**	NA
Microscopic hemorrhages (grade)	1 (±1.41)	1.93 (±1.65)	0.01 (±0.02)	0.72 (±0.86)	0.18	**0.008**
HA β-APP foci	0 (±0)	0.75 (±1.67)	0.01 (±0.02)	0.81 (±1.06)	0.09	**0.05**
NHA β-APP foci	1 (±1.53)	0.93 (±1.15)	0.59 (±0.67)	0.55 (±0.35)	0.89	0.83
Area of β-APP foci	2 (±2.65)	1.14 (±1.33)	5.15 (±6.96)	4.58 (±4.27)	0.79	0.60
HA fibrinogen foci	2 (±3.27)	4.11 (±5.24)	0 (±0)	0.79 (±1.11)	0.59	**0.003**
NHA fibrinogen foci	2.86 (±4.34)	0.79 (±1.87)	45.46 (±18.13)	38.17 (±13.55)	0.08	0.28

*SD, Standard deviation; NA, Not applicable; HA, hemorrhage associated; NHA, non-hemorrhage associated; Significant values are bolded*.

* *Differences based on Mann-Whitney U test*.

## Discussion

Lewallen et al. (1993) first described the malaria retinopathy-specific ophthalmologic findings in 1993. It has since been postulated that the pathological processes underlying malaria retinopathy reflect the pathological CM processes in the brain and that the presence and extent of visible retinopathy may not only predict the type and severity of central nervous system (CNS) involvement and disease outcome, but also contribute to our understanding of the pathogenesis of brain disease in CM (MacCormick et al., 2014). To date, however, there are no comparative studies of CNS and retinal lesions in the same patients that could confirm this supposition.

In this study, our comparisons focused on four pathological features: iRBC sequestration, perivascular hemorrhage, fibrinogen extravasation as an indicator of BBB and BRB breakdown, and axonal injury. In all malaria cases the capillaries and venules showed prominent iRBC sequestration that was equally prevalent in the brain and retina. We found that the number of ring hemorrhages in the brain positively correlated with clinical, gross and microscopic perivascular retinal hemorrhages. This finding is consistent with a previous clinicopathological study in Malawian children with fatal CM that showed correlation of the number of hemorrhages observed in the retina prior to death with those present at autopsy and with the number of hemorrhages present in the parietal lobe of the brain and the cerebellum (White et al., 2001).

Although a significant difference was found between CM1 and CM2 cases in the number of ring hemorrhages in the brain and gross retinal hemorrhages, this difference was not significant for clinical and microscopic retinal hemorrhages, since four CM1 patients that did not have detectable hemorrhages in the brain did have microscopic retinal hemorrhages. Thus, unlike the brain, perivascular retinal hemorrhages do occur in a subgroup of CM1 patients in association with heavy iRBC sequestration. The reason for the significant difference between gross retinal hemorrhages and brain hemorrhages may have included an underestimation of the hemorrhages against the dark choroid after formalin fixation. As well, ophthalmologic exams were carried out many hours prior to death and two patients showed a marked increase in retinal hemorrhages during this time leading to a discrepancy between clinical and microscopic hemorrhages.

Disruption of the BBB and BRB was present in all CM patients in the form of increased permeability to fibrinogen in association with either perivascular hemorrhages in the CM2 patients, or heavily parasitized microvessels in both CM1 and CM2 groups. In the CM2 group, the number of ring hemorrhage-associated foci of fibrinogen leakage in the brain positively correlated with the number of retinal hemorrhage-associated foci of fibrinogen extravasation, suggesting that there may be a similar mechanism of BBB and BRB disruption, presumably secondary to irreversible damage of the vascular endothelial lining. However, in the same group, microvessels with iRBC sequestration and fibrinogen leakage in the absence of hemorrhage were more frequent in the brain than the retina. Furthermore, in the retina the mean number of hemorrhage- and non-hemorrhage-associated foci of fibrinogen leakage was not significantly different between the CM1 and CM2 cases, unlike the brain where hemorrhage-associated fibrinogen leakage was consistently observed only in the CM2 cases. The mechanism of BBB and BRB disruption in heavily parasitized vessels is likely multifactorial and may reflect the effect on endothelial cells of several host and parasite factors, including, but not limited to direct contact with iRBC and parasite products released upon iRBC rupture, systemic and locally released cytokines produced by intravascular monocytes, and anoxia, which act alone or in concert to increase the permeability of the tight junctions.

The extent and frequency of axonal damage in the form of discrete foci of β-APP immunoreactive swollen or disrupted axons around ring hemorrhages in the CNS and perivascular retinal hemorrhages or diffuse foci of axonal damage in areas with prominent iRBC sequestration, correlated well in the brain and retina of affected individuals. However, in the retina, unlike the brain, the median number of hemorrhage- associated foci of axonal damage was not different between the CM1 and CM2 groups, since some perivascular hemorrhages still occurred in the retina in the CM1 group.

These results suggest that the retina and brain undergo similar disease processes during CM infection. This could be at least partly explained by the fact that both tissues have a common embryological origin, share certain types of resident cells and have similar morphological and functional endothelial barrier properties. The latter is reflected in the similar responses of BBB and BRB endothelial cells to cytokines, their expression of identical immunologically relevant surface molecules (Wang et al., 1995) and similarities in their interactions with circulating leukocytes (Greenwood and Calder, 1993; Wang et al., 1993). These common features may predispose the retina and brain to similar risk factors associated with the cerebral complications of *P. falciparum* infection.

This study points toward the convergence of important pathological processes in the brain and retina in pediatric CM. In addition, it indicates that while there is a difference in hemorrhage-associated pathology in the brains of CM1 and CM2 patients, this difference is not consistently observed in the retina. This could be due either to divergence in pathological processes observed in a few children (as numbers in the CM1 group are small), or it could be explained by tissue differences along a timeline of a common disease progression induced by individual differences in cytokine production. (Grau et al., 1987, 1989; Hunt and Grau, 2003). The cellular structure of the retina may also allow hemorrhages to occur earlier in the retina than in the brain. Work by Beare et al. (2004) showed an increased relative risk of death in patients that progressed at least two hemorrhage grades on repeated fundoscopic examinations. In murine models, microvascular pathology of the retina caused by malaria infection presents earlier in the overall disease process than it does in the brain (Chang-Ling et al., 1992). Perhaps a subset of our patients had not reached this stage of the disease process before their death. This explanation is supported by the similarity of non-hemorrhage associated pathology observed in both CM1 and CM2 patients. Fibrinogen leakage was only significantly different when hemorrhage associated and in the brain demonstrating the consistency with which fibrinogen leakage occurs in infected individuals in both the eyes and brain. The same results are true for axonal damage. While generally there is widespread damage in both the brains and retinas of CM1 and CM2 individuals, there was significantly more hemorrhage-associated axonal damage in CM2 patients. Fibrinogen leakage and axonal damage both reflect disruption of the blood-tissue barriers in retina and brain (Brown et al., 2001; Medana et al., 2002; Dorovini-Zis et al., 2011).

A minor limitation of our study is that fundoscopic examinations occurred at different time intervals prior to death in different patients. Funduscopic examinations performed repeatedly between patient admission up until the time of death could provide better understanding of the progression of the disease.

Previous studies have described the histopathology of the retina in CM and correlated these findings with the retinopathy that characterizes CM in children, thus providing the pathological substrate of the retinal lesions detected during life. Although autopsy studies provide a unique opportunity for the identification, analysis and classification of brain pathology in fatal CM, the presence, evolution and extent of CNS lesions cannot be accurately evaluated during life by the currently available clinical and laboratory tests. The present study is the first to directly compare the retinal and brain autopsy findings in the same group of children with fatal CM. Our results show important similarities in the type, frequency and extent of intra- and perivascular lesions, as well as BBB and BRB abnormalities between brain and retina. They also demonstrate differences between patients with and without microvascular pathology in the brain, but not in the retina, meaning that it may not be possible at present to distinguish CM1 and CM2 patients during life.

### Conflict of interest statement

The authors declare that the research was conducted in the absence of any commercial or financial relationships that could be construed as a potential conflict of interest.
